# Unconscious multisensory integration: behavioral and neural evidence from subliminal stimuli

**DOI:** 10.3389/fpsyg.2024.1396946

**Published:** 2024-07-18

**Authors:** Sergio Frumento, Greta Preatoni, Lauren Chee, Angelo Gemignani, Federico Ciotti, Danilo Menicucci, Stanisa Raspopovic

**Affiliations:** ^1^Department of Surgical, Medical, Molecular and Critical Area Pathology, University of Pisa, Pisa, Italy; ^2^Laboratory for Neuroengineering, Department of Health Sciences and Technology, Institute of Robotics and Intelligent Systems, ETH Zürich, Zürich, Switzerland; ^3^Clinical Psychology Branch, Azienda Ospedaliero-Universitaria Pisana, Pisa, Italy

**Keywords:** subliminal, multimodal integration, multisensory integration, unconscious integration, subthreshold, crossmodal integration, under threshold

## Abstract

**Introduction:**

The prevailing theories of consciousness consider the integration of different sensory stimuli as a key component for this phenomenon to rise on the brain level. Despite many theories and models have been proposed for multisensory integration between supraliminal stimuli (e.g., the optimal integration model), we do not know if multisensory integration occurs also for subliminal stimuli and what psychophysical mechanisms it follows.

**Methods:**

To investigate this, subjects were exposed to visual (Virtual Reality) and/or haptic stimuli (Electro-Cutaneous Stimulation) above or below their perceptual threshold. They had to discriminate, in a two-Alternative Forced Choice Task, the intensity of unimodal and/or bimodal stimuli. They were then asked to discriminate the sensory modality while recording their EEG responses.

**Results:**

We found evidence of multisensory integration for supraliminal condition, following the classical optimal model. Importantly, even for subliminal trials participant’s performances in the bimodal condition were significantly more accurate when discriminating the intensity of the stimulation. Moreover, significant differences emerged between unimodal and bimodal activity templates in parieto-temporal areas known for their integrative role.

**Discussion:**

These converging evidences - even if preliminary and needing confirmation from the collection of further data - suggest that subliminal multimodal stimuli can be integrated, thus filling a meaningful gap in the debate about the relationship between consciousness and multisensory integration.

## Introduction

Understanding how information is integrated and forms a conscious percept has been a challenge for scientists since decades: integration has been hypothesized to be a prerequisite of any conscious experience, a function of consciousness, or consciousness itself (Mudrik et al., 2014).

Indeed, many theories [labeled as *integration theories* of consciousness (Scott et al., 2018)] proposed that consciousness goes hand in hand with integration (Mudrik et al., 2014; Zher-Wen and Yu, 2023). For example, the global neuronal workspace theory (GNW) postulates that the conscious experience of a piece of information results from the integration of sensory stimuli processed by sensory as well as high-level areas (Dehaene et al., 2003)—though a recent version of GNW admitted the possibility of preconscious [i.e., “a transient […] state of activity in which information is potentially accessible, yet not accessed” (Dehaene et al., 2006)] multimodal integration to explain the early (<200 ms) activations at which “global brain activity splits between conscious and unconscious processing” (Sergent et al., 2021). The conscious access hypothesis (CAH) postulates that brain areas are independent and that “consciousness is needed to integrate multiple sensory inputs” (Baars, 2002). The integrated information theory (IIT) postulates that “consciousness requires both integration and differentiation” and even some “high-level cognitive performance such as judging whether a scene is congruous or incongruous […] lack integration and therefore are strictly unconscious” (Tononi et al., 2016). More broadly, the answer proposed for the consciousness/integration debate [e.g., that “globally integrated perceptual scenes […] can only be conscious” (Seth and Bayne, 2022)] has been labeled as one of the main factors differentiating each theory of consciousness from the others (Seth and Bayne, 2022).

The relationship between integration and consciousness has been investigated in studies showing that a subliminal stimulus facilitated the emergence of awareness of congruent supraliminal stimuli (Deroy et al., 2014). While noteworthy, this evidence does not clarify whether integration is necessary for stimulus awareness or it just makes it more likely. In fact, the integration theories of consciousness admit the possibility for a supraliminal stimulus to be integrated with a subliminal one. On the other hand, “only an experiment where both stimuli are unconsciously presented can truly probe unconscious multisensory integration” (Mudrik et al., 2014).

In this regard, only three recent studies administered subliminal stimuli coming from different sensory modalities during wakefulness (Zher-Wen and Yu, 2023). Faivre et al. (2014) found traces of multimodal integration for subliminal stimuli, but only after their previous supraliminal association (which suggests a determinant role of learning processes). Scott et al. (2018) measured if visual and auditory stimuli (i.e., words) could be integrated resulting in a priming effect (i.e., associative learning of stimulus pairs). Ching et al. (2019) checked whether an indicator of multimodal integration—the McGurk effect, i.e., an interference between unmatched auditory and visual clues of syllables’ pronunciation (Mcgurk and Macdonald, 1976)—could be observed even in response to subliminal clues. All these studies reported a significant effect of the multimodal subliminal stimulation; however, whether this effect could be interpreted in terms of multisensory subliminal integration is debated (Ching et al., 2019). Indeed, Ching et al. (2019) cast the doubt that the studies actually measured a mere interaction—rather than integration—between stimuli: they propose that the unimodal information may persist and influence later processes, without combining that information in a Gestalt (Ching et al., 2019).

Despite the noteworthy efforts profuse in these studies, the so-called integration theories of consciousness can be (dis)confirmed only by converging findings about a specific postulate (Seth and Bayne, 2022). This is the reason for which, beyond understanding if subliminal integration is possible, a comprehensive study should search for neuroimaging evidence of this integration and should check whether it follows a psychophysical model comparable to that of conscious integration. One of the most famous models regarding multisensory integration has been proposed by Ernst and Banks (2002), who proposed that humans integrate information similarly to a maximum-likelihood estimation (MLE). In their seminal work, they found that adults integrate multisensory stimuli performing a weighted estimation of the available sensory cues. This model has been validated for supraliminal stimuli, and today, it is still unknown whether it applies also to stimuli that are presented outside of conscious awareness (i.e., subliminal stimuli) or not [as reported for children (Gori et al., 2008; Negen et al., 2019)].

To check whether multimodal subliminal stimuli can undergo integration—and, if so, whether this is optimal or not—we applied the same psychophysical model from the seminal study of Ernst and Banks (2002) to one of the least studied combinations of subliminal sensory stimuli: visuo-haptic stimulation (Faivre et al., 2017). In a separate session involving EEG, we then investigated brain responses to each class of stimuli in terms of event-related potentials (ERPs).

## Materials and methods

In total, 12 healthy volunteers (7 males, 5 females) participated in the EEG session and 8 of them (5 males, 3 females) performed also the intensity discrimination experiment. All participants signed the informed consent. They were selected for having normal or corrected-to-normal vision and no history of sensory impairments. The experimental procedures were approved by the Institutional Ethics Committees of ETH Zurich (EK 2019-N-97) and carried out in accordance with the Declaration of Helsinki.

**Figure 1:**
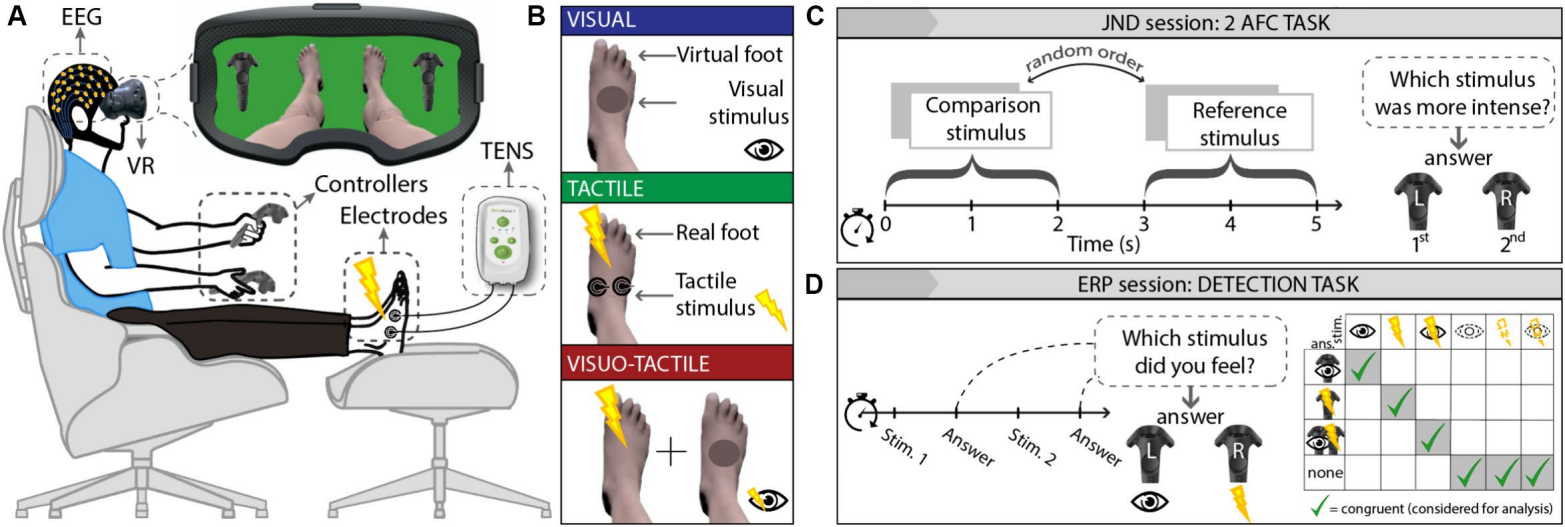
Experimental setting. **(A)** Set-up: participants wore a VR headset through which they were immersed in a scenario showing their virtual avatar’s lower body and two VR controllers corresponding to those held; two TENS electrodes were placed on participant’s right foot dorsum. For the ERP session, participants wore an EEG net. **(B)** Experimental conditions: visual (blue), tactile (green), and visuo-tactile (red). **(C)** JND session: participants had to judge which was the strongest among 2 consecutive stimuli by pressing the respective controller (a “1°” or “2°” icon appeared on the left and right controller). **(D)** ERP session: participants were asked to identify visual, tactile, or visuo-tactile nature of the trials by pressing the controller with the eye icon, the controller with the thunderbolt icon, or both, respectively. EEG, electroencephalography; VR, virtual reality; TENS, transcutaneous electrical nerve stimulation; solid contour lines = suprathreshold stimuli; dashed contour lines = subthreshold stimuli.

Healthy subjects were immersed in a virtual scenario (Figure 1A) by means of a VR headset. Visual stimuli consisted of gray circles appearing on the dorsum right foot; two TENS electrodes, applied on the same location of the right foot, delivered tactile stimuli consisting of electric pulses lasting 1 ms (Figure 1B). Volunteers participated in 2 experiments: 12 participants underwent an ERP (event-related potentials) session consisting of a detection task asking to signal the conscious perception of visual, tactile, or visuo-tactile stimuli (Figure 1D); 8 of them also underwent a JND (just-noticeable differences) session consisting of a two-alternative forced-choice (2-AFC) task asking to discriminate which was the strongest between two stimuli (Figure 1C). The order of sessions was randomized and carried out on different days distanced by 1 week.

During both experiments, a series of stimuli were administered, either unimodal (visual or tactile) or bimodal (visuo-tactile), either under or above the perceptual threshold. The visual stimulus consisted of a dark-red circle appearing on the right foot; the tactile stimulus consisted of an electrical current administered at the same point of the same foot; the visuo-tactile stimulus consisted of both the visual and the tactile stimuli administered simultaneously (Figure 1B). The conscious perception of the stimulus was manipulated by adjusting stimulus’ transparency (for visual stimuli) and by adjusting the pulsewidth and frequency of the electrical stimulation (for haptic stimuli). To find the perceptual threshold, a thorough characterization phase was performed immediately before each experimental session for each sensory mode and for each participant.

In this calibration phase (fully detailed in Supplementary Section 1.4), ramps of visual or tactile stimuli with increasing intensity were administered until the participant reported to have perceived a stimulus with above-chance confidence (i.e., detection threshold): the stimulus intensity was then averaged across at least 10 detection thresholds and increased or decreased by 15% to obtain subthreshold and suprathreshold stimuli. This criterion, derived from literature (Nierhaus et al., 2015) and preventing habituation effects (Rossi Sebastiano et al., 2022), was further validated in a subsequent check administering 15 subthreshold stimuli and 15 suprathreshold stimuli. This check was considered successful when at least 90% of the suprathreshold stimuli were felt, and at least 90% of the subthreshold stimuli were missed.

Importantly, the detection of each stimulus (regardless of it being administered above or below the calibrated threshold) was checked trial by trial, thus allowing the exclusion from the analyses of the incongruent stimuli (e.g., subliminal stimuli that were actually seen/felt). Indeed, participants were instructed to mark the stimulus as perceived by clicking the right or left VR controller according to the nature of percept (visual or tactile) or both for visuo-tactile stimuli. During both the calibration and the experimental phases, participants were further instructed to report the awareness of the stimuli when they perceived it with an above-chance confidence, i.e., not needing to be 100% sure: this approach guaranteed that stimuli marked as subliminal were, in fact, unconscious and not merely perceived with insufficient (though above-chance) confidence.

Evidence of multimodal integration was searched in terms of (1) significantly different accuracy in discriminating just-noticeable differences between consecutive trials and (2) significant differences between the EEG activity in response to unimodal or bimodal trials in a temporoparietal component that previous literature (Driver and Noesselt, 2008; Hidaka et al., 2015) indicated to account for visuo-tactile integration. Details about the statistical analyses implemented are fully reported in Supplementary Material: however, analysis files and EEG/JND data object of analysis are publicly shared at the Open Science Framework repository that can be reached at the link https://osf.io/5wsnk/.

### Just-noticeable differences (JNDs) session

Participants were asked to determine which was the strongest between the two trials, providing their answer through a VR controller (see Supplementary Material) in two conditions: comparing supraliminal trials and comparing subliminal trials. None of the stimuli (110 pairs of trials for participants) in the JND session had to be excluded from the analysis as the careful threshold calibration (see Supplementary Material) allowed all suprathreshold stimuli and none of the subthreshold ones to be perceived. We measured whether the distribution of answers followed a model of maximum-likelihood estimation (Ernst and Banks, 2002) (see Supplementary Material) and whether the accuracy in discriminating bimodal or unimodal trials was significantly different and higher than chance.

### Event-related potentials (ERPs) session

The ERP session consisted of the administration of 1,050 randomized trials that participants had to correctly detect and discriminate as being either tactile, visual, or visuo-tactile. Suprathreshold conditions consisted of 100 visual (V_SUPRA_), 100 tactile (T_SUPRA_), and 100 visuo-tactile (V_TSUPRA_) trials; subliminal conditions consisted of 250 visual (V_SUB_), 250 tactile (T_SUB_), and 250 visuo-tactile (VT_SUB_) trials. Stimuli were administered in a randomized order, with an intertrial interval (ITI) jittered between 1 and 2 s.

The EEG signals were acquired at 256 Hz with a 64-electrode cap, maintaining impedance below 5 kΩ (BE Plus LTM, EBNeuro, Florence, IT). Recorded EEG signals were submitted to the following preprocessing steps: (1) EEG signals were filtered in the 0.5–45-Hz filter (EEGLAB basic FIR filter); (2) EEG signals were visually scrolled for manual artifact identification, and any segment containing idiosyncratic artifacts (mostly due to small movements and temporary declines of signal quality) were highlighted and thus removed (EEGLAB); (3) noisy channels were identified, and their signal was substituted with signal obtained via spline interpolation (Junghofer et al., 2000); (4) EEG signals were submitted to the independent component analysis [Infomax (Bell and Sejnowski, 1995)] in order to remove ocular, cardiac, and muscular artifacts (Makeig et al., 1996) artifactual components were selected and removed based on a visual inspection of the component time course and its power spectrum, as well as on the analytic tools developed in the ICLabel toolbox (Pion-Tonachini et al., 2019) to support visual judgment examination; (5) the obtained EEG signals were finally re-referenced from the vertex to the common reference (Piarulli et al., 2010).

The scientific literature about EEG correlates of multisensory integration involves approaches [e.g., Global Field Power (Noel et al., 2019)], whole-scalp point-by-point analysis (Fossataro et al., 2023), ERP super-additivity (Ronga et al., 2021) that could be not perfectly suitable for the kind of data collected for the present study (i.e., correlates of very weak stimuli delivered slightly above or below the awareness threshold), as we better contextualized in the Supplementary Material (where we also provide ERP data obtained through more traditional approaches).

To check for traces of processing and integration of stimuli in the EEG recordings, we extracted via ICA (Bell and Sejnowski, 1995) four independent components temporally related to trials (Supplementary Figure S3) and the related activity templates (i.e., its time course averaged over trials).

The focus was directed to any component showing, at least for supraliminal stimuli, significant differences between the bimodal trials and both their unimodal correspondents while these were not significantly differing from each other. The strictness of these criteria aimed at excluding possible differences could be driven by the processing of single unimodal stimuli.

More in detail, for all subjects we selected trials for which the participant’s answer was congruent with the delivered stimulus/i (incongruent trials were 47% for V_SUPRA_, 35% for T_SUPRA_, 64% for VT_SUPRA_, 22% for V_SUB_, 4% for T_SUB_, 23% for VT_SUB_). ERP was thus extracted from the EEG signal based on the time location of congruent trials: each segment started from 100 ms before to 400 ms after each trial onset. To distinguish the putative temporoparietal component accounting for visuo-tactile integration (Driver and Noesselt, 2008; Hidaka et al., 2015) from components accounting for the processing of unimodal stimuli (Laurino et al., 2014), all ERP signals were concatenated and submitted to the independent component analysis (ICA).

The ICA-based ERP decomposition consisted of deriving a special combination of the different EEG channel signals that allow separating components originating from different brain sources (Jung et al., 2001). Thus, the ICA-based ERP decomposition modeled ERPs as the sum of temporally independent components (that is, with statistically independent time course) arising from distinct, spatially fixed, brain processes. Herein, a group-based ICA decomposition (Himberg et al., 2004; Menicucci et al., 2014) was performed by applying the Infomax ICA algorithm (Bell and Sejnowski, 1995) on the concatenated ERPs of all trial types and subjects. This approach implied the assumption that all subjects had comparable brain components and that the stimulus awareness (subliminal or supraliminal) modulated the time course of components but did not affect their scalp distribution (Jung et al., 2001).

The number of underlying components was determined based on a preliminary principal component analysis by retaining components explaining 95% of the total ERP variance. On this basis, four components were retained and among them, the temporoparietal component was selected. The scalp distribution (i.e., the contribution to the potentials recorded at each scalp channel) and the time course of independent components were provided by ICA as corresponding to the demixing matrix and to the activation time series, respectively. Finally, as ICA was performed at the group level with all trials and subjects ERPs concatenated together, from each component activation time series we derived the activity templates showing the average component activity for each stimulus type and subject.

For the selected component, we compared (subject-based paired *t*-test) bimodal and unimodal activity templates for both subliminal and supraliminal modalities. Searching for traces of multimodal integration, we checked for the latencies at which bimodal activity templates were significantly different from their unimodal correspondents simultaneously, while the unimodal activity templates were not significantly differing from each other: in addition, to preserve both the sensitivity and the reliability of the statistical analysis, we considered worth of being interpreted as positive results only consecutively significant latencies with a total duration of at least 12 ms (i.e., three consecutive samples). For the sake of completeness, the same comparisons were performed for the other components extracted and are reported in Supplementary Figure S4. Finally, to provide further information about the brain origin of the component, we used the EEGLAB Dipfit 4.3 plugin to estimate the equivalent current dipoles adjusted by means of the boundary element model (BEM) of the head (Bocharov et al., 2020).

This approach stands on the assumption that local cortical connections are characterized by a much higher density than longer range ones; this premised, it can be assumed that synchronous coupling of neuronal activity isolated by ICA typically occurs within a single brain area. The resulting scalp maps can highly resemble the projection of a single equivalent dipole or a bilaterally symmetric pair of dipoles and may thus represent a projection of activity from one patch—or two symmetric patches—of the cortex.

The combination of all the above-mentioned criteria implies that significant differences possibly observed in one or more components could be more reliably interpreted as a marker of subliminal multisensory integration if (1) they appeared in the supraliminal conditions too, for which there is robust evidence that multisensory integration occurs, (2) the topography is compatible with multisensory hubs reported in the scientific literature, (3) their duration of at least 12 ms reasonably rules out the occurrence of mere coincidences, and (4) the absence of simultaneous significant differences between the two unimodal conditions reasonably rules out that the bimodal activity template is over-representing the processing of one kind of stimulus only.

## Results

### Just-noticeable differences (JNDs)

**Figure 2:**
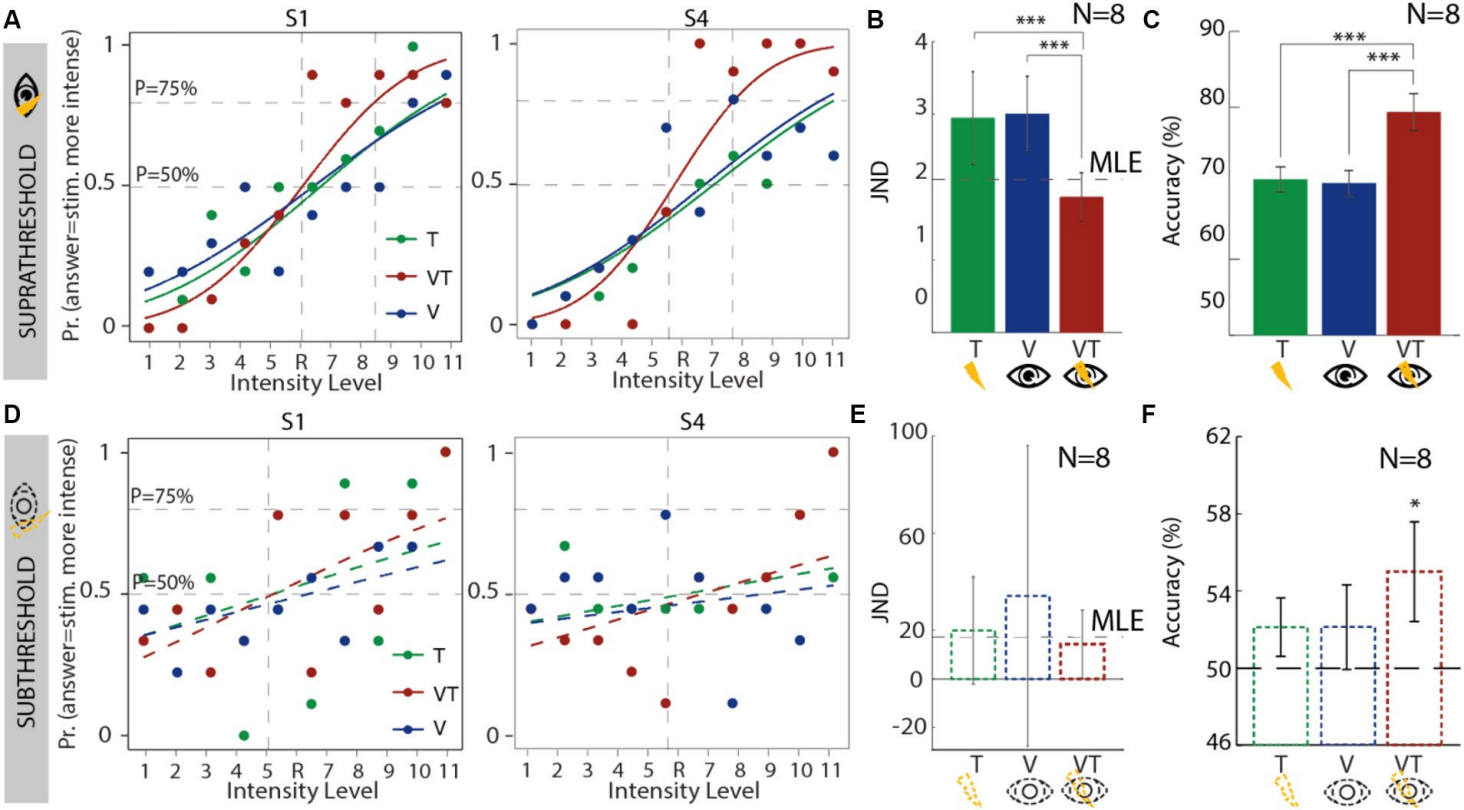
Results for the JND session for all conditions: touch (green), visual (blue), and visuo-tactile (red). **(A–C)** Results for the suprathreshold condition: **(A)** psychometric curves of two exemplary subjects; **(B)** JNDs for all subjects. Bar plots represent mean and CI. The dashed horizontal line represents the predicted behavior following the MLE model; **(C)** accuracy. Results are presented as mean ± standard error of the mean. **(D–F)** Results for the subthreshold condition: **(D)** psychometric curves of two exemplary subjects; **(E)** JNDs for all subjects. Bar plots represent mean and standard error of the mean. The dashed horizontal line represents the predicted behavior following the MLE model; **(F)** accuracy: dashed line represents chance level. Results are presented as mean ± standard error of the mean. T, tactile; V, visual; VT, visuo-tactile; MLE, maximum-likelihood estimation.

For the suprathreshold condition (Figure 2A and Supplementary Figure S1A), the pseudo-R2 (see Supplementary Material) indicated a high Goodness of fit ( $R_{L}^{2}$ = 0.78), meaning that the model fitted well the experimental results. The visual JND (JND_V_SUPRA_ = 3.006) was not statistically different (*p* = 0.62) from the tactile JND (JND_T_SUPRA_ = 2.95), indicating that the values were rescaled correctly to allow similar weights (see Supplementary Material) and avoid having a dominant sensory modality (Supplementary Figure S2). As predicted by the MLE model, the bimodal condition (JND_VT_SUPRA_ = 1.86) was significantly smaller compared to both the tactile (*p* = 0.006, power = 0.97, es = 3.81) and visual JND (*p* = 0.0007, power = 0.99, *effect size = 5.02*) (Figure 2B). Moreover, the bimodal JND was the most similar to the predicted behavior from the MLE model (JND_MLE_SUPRA_ = 2.1).

For the subliminal condition (Figure 2D and Supplementary Figure S1B), the pseudo-R2 indicated a very low Goodness of fit ( $R_{L}^{2}$ = 0.23), meaning that the model could not fit sufficiently well the experimental data. Moreover, none of the experimental conditions differed (*p* > 0.5) in their JND (JND_T_SUB_ = 19.98, JND_V_SUB_ = 34.2, JND_VT_SUB_ = 14.3, JND_MLE_SUB_ = 17.26) (Figure 2E).

We then explored how well the subjects performed in each condition, hence how accurate they were in indicating which was the most intense stimulus and if a bimodal stimulation would allow a better performance. In the suprathreshold condition (Figure 2C), the bimodal performance (ACC_VT_SUPRA_ = 79.3%) was significantly higher than the tactile one (ACC_T_SUPRA_ = 70.5%, *p* = 0.03, power = 0.97, *effect size = 1.44*) and the visual one (ACC_V_SUPRA_ = 70%, *p* = 0.03, power = 0.98, *effect size = 1.52*). In the subthreshold condition, the accuracies were not significantly different from each other (*p* = 0.67). However, when comparing these to the chance level (50%), the bimodal condition had a significantly higher accuracy (ACC_VT_SUB_ = 55%, *p* = 0.04, power = 0.53, *effect size = 0.7*) (Figure 2E).

### Event-related potentials (ERPs)

A first analysis of ERPs compared unimodal to bimodal stimuli finding no significant differences worth being interpreted as signs of multimodal integration: details about this analysis are reported in the Supplementary Sections 2.2, 2.3, and a representative selection of these results is visible in Supplementary Figures S5–S8.

For what concerns the ICA-based ERP decomposition, three out of the four extracted components were excluded due to the absence of statistical significance across the different conditions, as illustrated in Supplementary Figure S4. This finding precludes their interpretation as a reliable marker of multisensory integration. In fact, Supplementary Figure S4 shows that Component A (visual component exhibiting a late positive peak for all visual supraliminal stimulations), Component B (central areas that isolate the P300), and Component C (left temporoparietal area) lacked differences interpretable as multimodal integration (Supplementary Figure S4). Importantly, these differences were missing also in supraliminal conditions, for which behavioral correlates indicated that bimodal stimuli were in fact integrated [coherently with the scientific literature (Ernst and Banks, 2002)].

**Figure 3:**
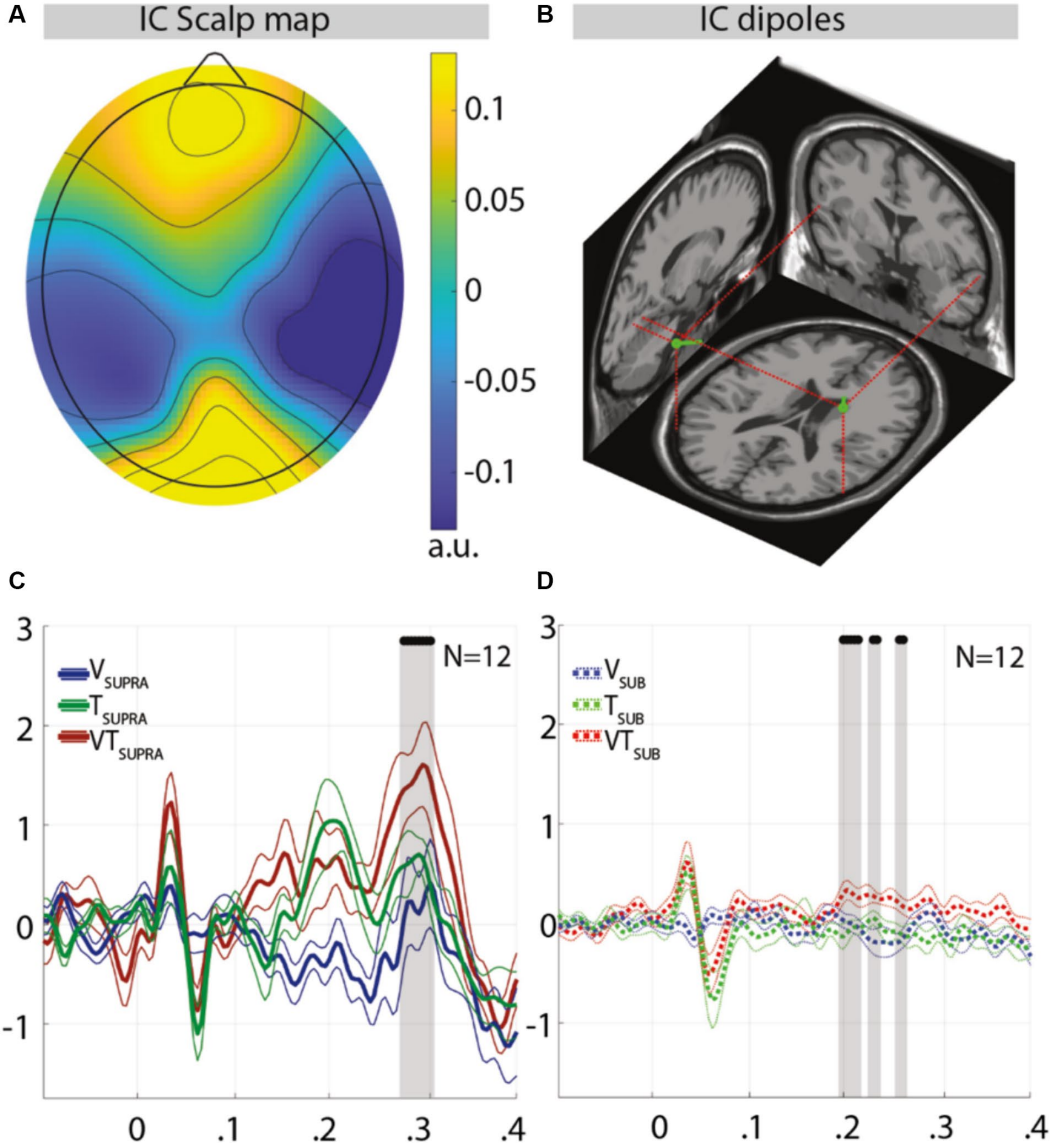
Topography and activity templates of the integrative temporoparietal independent component in response to supraliminal or subliminal, unimodal, or bimodal trials. **(A)** Component scalp topography and the related dipole locations **(B)**. **(C)** Activity templates for supraliminal trials and subliminal trials **(D)**. Thicker lines (solid or dashed for supraliminal or subliminal trials, respectively) represent the group mean; thinner ones represent the group mean ± the standard error of the mean (SEM); the black dots represent latencies at which bimodal trials significantly (*p* < 0.05) differ from both unimodal trials simultaneously, while these latter were not significantly differing from each other; a.u., arbitrary units. The earlier component appearing 50 ms after stimulus onset is driven by the tactile stimulus, coherently with previous results comparing ERPs of subliminal and supraliminal tactile stimuli (Nierhaus et al., 2015); the time windows of the latter components (190–260 ms for subliminal conditions; 275–300 ms for supraliminal conditions) are temporally complementary and coherent with the timing (250–300 ms) of the processes that differentiate conscious from unconscious processing (Sergent et al., 2021).

On the other hand, a temporoparietal component (Figure 3A) showed significant differences, coherently to the hypothesis that in these regions visuo-tactile integration would occur (Driver and Noesselt, 2008; Hidaka et al., 2015). Figure 3B shows the scalp localization and the estimated current dipoles of this component. The dipole locations were compatible with sources placed in Brodmann areas 37 and 19, visual areas known for their associative functions (e.g., their lesion impairs the ability to compute a semantic representation of stimuli) (Race and Hillis, 2015). For what concerns the supraliminal stimuli, differences in this component’s activity templates were located around latencies of 300 ms, with bimodal trials producing higher positive responses. For what concerns the subliminal stimuli, differences started from 200 ms (a latency at which sensory stimuli evoke specific responses even if delivered during sleep) (Laurino et al., 2014) and highlighted a steady positivity differentiating bimodal-related activity template from the corresponding unimodal ones.

Considering visual, tactile, and visuo-tactile trials separately, the comparisons between supraliminal and subliminal-related activity templates (Supplementary Figure S3) showed much of the differences at latencies after 200 ms, with supraliminal responses exhibiting higher positivity compared to subliminal ones peaking at 300 ms *circa*. This higher positivity is coherent with the scientific literature describing P300 responses to consciously detected stimuli (Pfabigan et al., 2014), though is debated whether P300 should be considered a correlate of conscious perception or detection report (Tsuchiya et al., 2015).

The methodological reasons (e.g., weak intensity of stimuli) that could have possibly resulted in a lack of replication between the two analyses (ERP and ICA-based ERP decomposition) are deepened in the Supplementary Sections 2.2, 2.3.

## Discussion

The results reported in the present study show both behavioral and neuroimaging significant differences between bimodal (visuo-tactile) and unimodal (visual or tactile) stimuli, even when these stimuli were subliminal.

The supraliminal conditions of the JND session replicated the results initially reported in the seminal paper by Ernst and Banks (2002): bimodal stimuli were integrated following the expected maximum-likelihood estimation (MLE) model and were discriminated with an accuracy significantly higher than their unimodal counterparts (Figure 2C).

On the other side, the subliminal conditions did not show a significantly higher accuracy for the discrimination of bimodal (with respect to unimodal) stimuli, nor an adherence to the MLE model; however, participants showed significantly higher-than-chance performance in discriminating just-noticeable differences (JNDs) between subliminal stimuli only if these were bimodal (Figure 2F).

Should these significant differences be interpreted as a behavioral correlate of multisensory integration, even if they do not perfectly match the results reported for supraliminal conditions? The answer to this question is debatable, as it can vary depending on what we mean by “multisensory integration”; however, authoritative definitions of multisensory integration [e.g., “the process by which inputs from two or more senses are combined to form a product that is distinct from […] the components from which it is created,” and “a statistically significant difference between the response evoked by a cross-modal combination of stimuli and that evoked by the most effective of its components individually” (Stein et al., 2014)] fit with the evidence shown for subliminal conditions (i.e., a significantly higher-than-chance performance in discriminating bimodal stimuli, but not unimodal ones). Coherently with this definition, it is worth noting that multisensory integration does not always follow the model described by Ernst and Banks (2002): for example, the results they obtained in adults did not match those later obtained in children (Gori et al., 2008; Negen et al., 2019). This was not interpreted as an inability of children to integrate multisensory stimuli, but rather as the result of a task-dependent strategy—that changes across development—attributing a different weight to sensory modes (Gori et al., 2008).

This all considered, the significantly higher-than-chance accuracy in discriminating subliminal bimodal stimuli—but not unimodal ones—is reasonably interpretable as a clue of multisensory integration, even if different from that occurring for supraliminal stimuli.

The ICA-based ERP decomposition resulted in four components (the topography of which is shown in Supplementary Figures S3A–D) for which significant differences are reported when comparing each class of supraliminal stimuli with its subliminal counterpart (Supplementary Figure S3), coherently with the scientific literature (Nierhaus et al., 2015). Multimodal integration was expected to result in significant differences between bimodal (i.e., visuo-tactile) stimuli and both their unimodal (i.e., visual or tactile) counterparts while these were not significantly differing from each other: the meeting of all these conditions can reasonably guarantee that significant differences between bimodal and unimodal stimuli are not solely attributable to the influence of the more dominant unimodal stimulus. Coherently with the scientific literature (Pfabigan et al., 2014) and with the results of the JND session, these significant differences were found in one of the four components (Figure 3A); while this was expected in comparing supraliminal stimuli (Figure 3C), their presence in comparing subliminal stimuli (Figure 3D) represents an unprecedented result. Interestingly, the latencies at which these significant differences occurred (i.e., 190–260 ms for subliminal conditions; 275–300 ms for supraliminal conditions) seem to be complementary rather than overlapping, suggesting that the underlying processing was qualitatively different. This could mean that the brain processes underlying the multisensory integration of subliminal stimuli are not just a weaker version of those related to the multisensory integration of supraliminal stimuli: the “ignition” —i.e., “a sudden non-linear transition toward a state of globally increased brain activity” (Dehaene et al., 2003) thought to result in conscious access to stimuli (Seth and Bayne, 2022)—could consist of recurrent processing (not recurring enough, in the case of subliminal stimuli) rather than of a broadcast of information across distant areas. Interestingly, the temporal window of ignition (250–300 ms post-stimulus) estimated in a recent study (Sergent et al., 2021) begins at the end of the temporal window that we interpret as an EEG correlate of subliminal multisensory integration, allowing us to hypothesize that ignition follows a subliminal multisensory integration whose accuracy, while sub-optimal, could represent “an early evaluator of sensory coherence” (Ching et al., 2019).

Finally, while the topographical distribution of components showing no significant differences included primary sensory areas (e.g., Supplementary Figures S3A, S4A), the source estimated for the parieto-temporal component showing significant differences (Figure 3B) indicated the involvement of associative visual areas (i.e., Brodmann areas 37 and 19) (Driver and Noesselt, 2008; Hidaka et al., 2015)—coherently with our interpretation of differences in this component as a correlate of multisensory integration. Our findings provide supportive evidence for the hypothesis that visual and tactile stimuli undergo multimodal integration, even when presented subliminally. However, we acknowledge that further analysis is needed to decisively determine whether the responses to bimodal stimuli are distinctly different from the mere additive effects of unimodal stimuli.

## Conclusion

The present study introduces a novel paradigm to investigate both behavioral and neuroimaging correlates of the integration of bimodal stimuli that are both subliminal, thus testing a postulate of integration theories of consciousness (Scott et al., 2018) and filling a noteworthy gap in the scientific literature—so far reporting only behavioral correlates of multimodal subliminal integration or the integration of a subliminal stimulus with a supraliminal one (Zher-Wen and Yu, 2023).

The relatively small sample involved—although at least double that of Ernst and Banks’ seminal paper (Ernst and Banks, 2002)—implies caution in generalizing the present data. Nevertheless, with respect to the criticisms typically raised in the research lines involving subliminal stimulation (Wiens, 2007; Baroni et al., 2021; Frumento et al., 2021, 2022; Cesari et al., 2023) and multisensory subliminal integration (Mudrik et al., 2014), the methodological robustness of the present study was guaranteed by (1) the trial-by-trial assessment of stimulus detection, (2) the fine calibration measured (and furtherly checked) for each sensory mode, for each participant, before each experiment, (3) the exclusion of incongruent stimuli from analysis, and (4) the subliminal administration of stimuli coming from different sensory modes (tactile and visual).

The results show significant differences between bimodal and unimodal stimuli in both behavioral and neuroimaging correlates. This evidence supports each other in suggesting that conscious awareness is not needed to integrate stimuli coming from different sensory modes. While there is not a universally agreed agreement on what multisensory integration is, the reported evidences fit with authoritative definitions (Stein et al., 2014). To this regard, it is worth noting that each of the reported results, taken individually, could be not considered a definitive proof of subliminal multimodal integration: as an example, the observation that unimodal/bimodal differences are earlier for subliminal than for supraliminal stimuli could be interpretable as a confirmation of the relevance of recurring processes for awareness proposed by some integration theories of consciousness (Scott et al., 2018). Similarly, even if the significant differences between the JND and EEG correlates of unimodal and bimodal subliminal stimuli contrast some IIT postulates [e.g., “consciousness requires both integration and differentiation”; “high-level cognitive performance such as judging whether a scene is congruous or incongruous […] lack integration and therefore are strictly unconscious (Tononi et al., 2016)”], the comparisons between subliminal and supraliminal stimuli (Supplementary Figures S3, S5–S8) replicate those expected by IIT (Nierhaus et al., 2015).

However, the convergence of behavioral and neuroimaging correlates of subliminal stimulations and their coherency with the correlates of supraliminal integration can reasonably be interpreted, as a whole, as convincing evidence that subliminal multimodal integration is possible. Indeed, our brain can not only integrate multimodal stimuli we are not aware of, but it can also trick ourselves into believing to be randomly guessing in a cognitive task (e.g., discriminating just-noticeable differences between subliminal stimuli) while in fact our accuracy is significantly higher than chance. Is our consciousness just a passive spectator who deludes himself about being relevant for higher-order functions? To answer this question, it is worth looking at the other side of the coin, i.e., comparing subliminal stimuli with their supraliminal counterparts. In fact, supraliminal multimodal integration showed qualitatively different correlates with respect to those of subliminal multimodal integration. In particular, the statistical models describing the integration of bimodal trials in the JND session differed depending on their stimulation being supraliminal or subliminal: the former followed the model of maximum-likelihood estimation [replicating the seminal experiment by Ernst and Banks (2002)], while the latter did not. However, when the stimuli were subliminal, a form of integration still occurred. Indeed, we observed a higher-than-chance accuracy only for bimodal trials, which was not as accurate as for supraliminal trials—similar to what was demonstrated in children, who are nevertheless thought to integrate multisensory stimuli (Gori et al., 2008; Negen et al., 2019). This form of integration, if confirmed also in modality-independent integrative regions (Setti et al., 2023) and/or in superior colliculus (Tamietto et al., 2010), could underlie phenomena such as obstacle avoidance in blindsight (de Gelder et al., 2008). At least in the context of this specific task, the role of consciousness resembles that of an optimal integrator refining an accuracy that already resulted to be significantly higher than chance at an unconscious level. Further studies are needed to test the hypothesis that consciousness is an optimal integrator: in fact, the stimuli administered in the present study differed not only for being supraliminal or subliminal but also for their absolute intensity. To rule out the possibility that optimal integration occurred only for supraliminal stimuli because of their higher absolute intensity, a replication of the JND session is needed administering stimuli the intensity of which falls exactly on the calibrated threshold (rather than slightly below or above it), so that *circa* half of them should result subliminal.

In conclusion, the present study is the first to describe the integration of bimodal stimuli occurring even if they are subliminal, thus opening impactful clinical and theoretical implications. The former could pave the way for the implementation of subthreshold stimulations in rehabilitation neuroprostheses (Raspopovic, 2020; Preatoni et al., 2021; Chee et al., 2022), enhancing their acceptability (Preatoni et al., 2021; Valle et al., 2021; Cesari et al., 2024) while maintaining a comparable efficacy. In this regard, further studies are needed to investigate the integration of a subliminal stimulus with a supraliminal one and to test the clinical applicability of the results [e.g., in clinical populations susceptible to interoceptive specificities (Alfì et al., 2023; Cipriani et al., 2024)].

For what concerns the theoretical implications, the significant differences between unimodal and bimodal subliminal stimuli observed in both the JND and the EEG sessions converge to suggest that multimodal integration is related to stimulus awareness but not “needed” (Baars, 2002) for its occurrence: however, this evidence—while contradicting an assumption shared by the so-called integration theories of consciousness (Scott et al., 2018)—does not represent a disconfirmation of each of these theories as a whole (on the contrary, as detailed previously in this chapter, it confirms other points). It is also worth noting that these results are currently based on an inevitably limited amount of data and should thus be interpreted with caution until they are corroborated by future research able to put into practice the improvements proposed in the next chapter.

The whole debate about consciousness was centered for decades on the idea that stimulus awareness and integration are necessarily interdependent (Mudrik et al., 2014): the theories based on this assumption should evolve to fit with the evidence coming from the present study.

## Limitations of the study

Some noteworthy methodological issues are known to affect the scientific literature concerning subliminal stimuli and the interpretation of their behavioral or neuroimaging correlates (Wiens, 2007; Baroni et al., 2021; Frumento et al., 2021, 2022; Zher-Wen and Yu, 2023).

The most relevant problem typically concerns the meaning attributed to the term “subliminal,” and the reliability of methods used to label stimuli as such (Wiens, 2007; Zher-Wen and Yu, 2023). In fact, the mere calibration of a perceptual threshold is not sufficient to guarantee that all stimuli below this threshold will be not consciously perceived (nor that all stimuli above this threshold will be consciously perceived) (Frumento et al., 2022): in addition, the intensity calibrated for tactile stimuli and for visual stimuli to result subliminal could sum up and induce a conscious experience of the bimodal stimulus.

The most reliable method to assess stimulus detection consists in a trial-by-trial report (not necessarily verbal) of its awareness (Wiens, 2006), but this procedure implies decisional and motor processes the correlates of which could be misinterpreted as a clue of multisensory integration [the reason why no-report paradigms are preferable in studies primarily aimed at comparing neural correlates of conscious and non-conscious stimuli (Kapoor et al., 2022)]. However, the main aim of the present study was to compare possible differences in behavioral and neuroimaging correlates of bimodal or unimodal stimuli that are subliminal, i.e., that do not imply any decisional or motor process to be labeled as subliminal (indeed, the lack of a report is the probe of their subliminality): the trial-by-trial assessment of stimulus detection allowed to exclude incongruent stimuli from analysis (see Figure 1D), thus eluding the possibility that supposed-to-be-subliminal bimodal stimuli were in fact consciously perceived because of a possible summation effect of the unimodal subliminal thresholds.

While adopting a robust methodology to calibrate stimuli intensity and to assess their detection, this procedure is necessarily based on subjective reports and can thus be affected by participant’s psychological variables (e.g., interpretation of instructions, level of attention, and compliance with the experimenter). Nevertheless, subjective reports represent the best assessment technique for experiments centered on the administration of subliminal stimuli (Wiens, 2007). To counterbalance the possible issues inevitably coming with this procedure, many measures were taken: intensity calibration needed to pass a rigid check-proof of its efficacy before each experiment; participants were instructed to mark stimuli as perceived when their confidence in the response was above chance, thus inducing the adoption of conservative criteria; stimulus detection was assessed on a trial-by-trial basis, following methodological indications coming from the scientific literature concerning subliminal stimulation (Baroni et al., 2021; Frumento et al., 2021, 2022; Frumento et al., 2022); a break allowed participants to restore their attention level and to maintain it constant during the whole experiment. Furthermore, we acknowledge that the sample size in our study, while yielding effect sizes indicative of strong effects, is relatively modest. Indeed, even though our sample size is placed within the generally employed standard in the field (Ernst and Banks, 2002; Laurino et al., 2014; Dadarlat et al., 2015; Marasco et al., 2018; Risso et al., 2019, 2022), we understand that this limits the extent to which our findings can be generalized. Future studies with larger and more diverse populations are warranted to replicate and potentially expand upon our results, ensuring robustness and wider applicability within the field of subliminal stimulation research.

## Data availability statement

The datasets presented in this study can be found in online repositories. The names of the repository/repositories and accession number(s) can be found at: https://osf.io/5wsnk/.

## Ethics statement

The studies involving humans were approved by Eidgenössische Technische Hochschule Zürich (EK 2019-N-97). The studies were conducted in accordance with the local legislation and institutional requirements. The participants provided their written informed consent to participate in this study.

## Author contributions

SF: Writing – original draft, Visualization, Methodology, Investigation, Formal analysis, Data curation, Conceptualization. GP: Writing – original draft, Visualization, Methodology, Investigation, Formal analysis, Data curation, Conceptualization. LC: Writing – review & editing, Validation, Software, Conceptualization. AG: Writing – review & editing, Supervision, Resources, Funding acquisition. FC: Writing – review & editing, Visualization, Formal analysis, Data curation. DM: Writing – review & editing, Visualization, Validation, Supervision, Methodology, Formal analysis, Data curation, Conceptualization. SR: Writing – review & editing, Supervision, Resources, Project administration, Methodology, Funding acquisition, Conceptualization.
