# Supplementary material for: Unconscious multisensory integration: behavioral and neural evidence from subliminal stimuli
Source: Front Psychol. 2024 Jul 18;15:1396946. doi: 10.3389/fpsyg.2024.1396946 (PMC11291458; doi:10.3389/fpsyg.2024.1396946)
Supplement: Supplementary file 1 [file Data_Sheet_1.docx]

# Supplemental Inventory

Supplementary Materials and methods

Figure S1

Figure S2

Figure S3

Figure S4

Figure S5

Figure S6

Figure S7

Figure S8

# 1. Supplementary material and methods

## 1.1 Participants

Twelve healthy volunteers (7 males, 5 females) participated in the ERP session and 8 of them (5 males, 3 females) performed also the JND experiment. All participants signed the informed consent. They were selected for having normal or corrected-to-normal vision and no history of sensory impairments. The experimental procedures were approved by the Institutional Ethics Committees of ETH Zurich (EK 2019-N-97).

## 1.2 Experimental settings

The resolution of the HTC Vive headset was 1440 x 1600 pixels per eye (2880 x 1600 pixels combined) and the refresh rate was 90 Hz). Both the EEG recording system and the TENS stimulator were unplugged from the electric current just before starting each experiment, relying on their batteries: this prevented the recording of electrical artifacts in the EEG tracks related to the electro-tactile stimulation.

## 1.3 Experimental protocol

Participants underwent two experiments in two different sessions; the JND session aimed at studying Just Noticeable Differences (JND) between both supraliminal and subliminal stimuli, the ERP session aimed at investigating cortical processes underlying multimodal integration by analyzing Event Related Potentials (ERPs) from EEG recordings. For each participant, experiment order was randomized, and sessions were organized at least one week and no more than 1 month far from each other.

In both experiments, participants were seated on a comfortable chair facing a support for their legs (Figure 1A): they were asked to take off shoes and socks of both feet and to lie their legs on a stool. They wore a VR headset (HTC Vive, Valve Corporation, USA), and, only for the ERP session, an EEG electrode cap (EBNeuro, Italy). They also handled two VR controllers (one for each hand) and had two TENS electrodes on their right foot. During both experiments, they saw their feet in first-person view within the VR scenarios (designed in a Unity3D engine, Unity Technologies, United States), receiving tactile and/or visual stimuli on the central dorsum of the right foot. Tactile stimuli consisted of an electrical current applied by a TENS stimulator (RehaMove, HASOMED GmbH, Germany) and two TENS electrodes (circle Pads, Ø = 25mm, TensCare, England) placed on the dorsum on the right foot; visual stimuli consisted of a dark circular spot appearing in the same location of the ‘virtual’ right foot (Figure 1B).

## 1.4 Calibration of stimuli

A calibration procedure was prepared in order to estimate the subjective threshold for both visual and tactile stimuli to be perceived.

As a first step, participants were administered a test stimulation to make them acquainted with tactile and visual stimuli. To determine the subjective detection threshold (i.e., the intensity «at which participants claim not to be able to discriminate perceptual information at better than a chance level»^1^), each participant underwent two calibration phases: one for the visual stimulus and one for the tactile stimulus.

For the tactile calibration, an initial ramp of stimulation with fixed pulsewidth (300ms) and frequency (50Hz) was performed to find a comfortable amplitude. With this value, 10 ramps of stimulation with fixed frequency and pulsewidth modulation were delivered to the subject, who was instructed to verbally report as soon as he felt a sensation even if she/he was not sure (above chance certainty). The mean of these ramps was considered as the subject’s perceptual threshold.

The visual calibration consisted of 10 ramps of decreasing transparency of a dark-red circle flashing for 1ms on the same location where the haptic stimulation was delivered. To confirm the reliability of this procedure, participants underwent a calibration check consisting in the administration of 15 trials per each of the 6 possible conditions: Visual Suprathreshold (V_SUPRA_) and Subthreshold (V_SUB_), Tactile Suprathreshold (T_SUPRA_) and Subthreshold (T_SUB_), Visuo-Tactile Suprathreshold (VT_SUPRA_) and Subthreshold (VT_SUB_).

To check for the efficacy of the calibration procedures, each participant received 30 stimuli with the parameters found during the calibration (15 sub-threshold, 15 supra-threshold): the participants were informed about the kind of stimulus that they were going to receive (either tactile or visual, both subthreshold and suprathreshold), and were instructed to click the controller as soon as the stimulus was detected. The calibration was considered successful if subjects identified at least 90% of the suprathreshold stimuli, and missed (correctly) at least 90% of the subthreshold stimuli. If these percentages were not reached, a new cycle of calibration was done until the achievement of satisfactory rates. The final suprathreshold and subthreshold stimuli obtained at the end of this whole procedure were supposed to be respectively supraliminal (i.e., reported for conscious perception during a detection task) and subliminal (i.e., not reported during a detection task, thus not consciously perceived). However, some suprathreshold stimuli could be missed (thus resulting subliminal), as well as some subthreshold stimuli could be perceived (thus resulting supraliminal): in the analysis of results, we considered only trials that resulted to be coherent with the intensity of stimulation (e.g., subthreshold stimuli that were not consciously perceived during a detection task).

## 1.5 Calibration procedure for JND

For the visual stimuli, the range of intensities represented the percentage of opacity of the stimulus shown on the foot of the subject. To decide the boundaries of minimum and maximum opacity, the following procedure took place: the subthreshold maximum opacity represented the 85% of the perceptual threshold and the minimum was 0%; the suprathreshold maximum opacity consisted in 100% (no opacity) and the minimum in the 50%.

For the tactile stimuli, the range of intensities represented the current injected. The minimum and maximum for the subthreshold condition followed the same approach as for the visual stimuli (0-85% of perceptual threshold). For the suprathreshold, since there was not an intensity comparable to 100%, we performed additional 10 ramps of stimulation with fixed frequency at 50Hz and amplitude found for the perceptual threshold, while increasing the pulsewidth. Subjects were asked to report when they felt a sensation of intensity 2 (minimum) and 8 (maximum). The average of the 10 values for the low intensity and of the 10 values for the high intensity were chosen as minimum and maximum. For the bimodal conditions (VT_SUPRA_ and VT_SUB_), the intensities of the visual and tactile stimuli were congruent.

# 2. Experimental sessions

## 2.1 JND (Just Noticeable Differences) session

Subjects received unimodal (visual or tactile) or bimodal (visuo-tactile) stimuli (Figure 1B), either above or below the averaged subjective threshold, resulting in a total of 6 conditions: V_SUPRA_, V_SUB_, T_SUPRA_, T_SUB_, VT_SUPRA_ and VT_SUB_. To apply the same mathematical procedures as in Ernst and Banks^2^, subjects were asked to perform a two-alternatives forced-choice (2-AFC) task where they had to discriminate which of two consecutive trials had the strongest intensity (Figure 1C). Therefore, to obtain the range of minimum/maximum intensities for the visual and/or tactile stimuli, a further calibration step had to take place.

Within these ranges, 11 equally-spaced stimuli were extracted for each of the six conditions. For each trial, an unchanging reference stimulus (corresponding to the middle one of the 11 extracted) was presented sequentially with another stimulus randomly chosen from the extracted set. The intra-order of couples (i.e., the order between reference and comparison stimulus) was randomized and each couple was presented 10 times, resulting in a total of 110 trials for each condition.

Participants could answer whether the stimulus having the strongest intensity was the first or the second one between the two administered (each followed by a grey screen to indicate the end of the stimulation) by pressing the VR controller corresponding to that shown in the virtual environment, marked with a “1°” and a “2°”. These responses were fitted with a cumulative normal probability distribution with the MixedPsy library^3^ to obtain a psychometric function, which represented the probability of judging the comparison stimulus to be more intense than the reference one. For each psychometric curve, we computed the point of subjective equality (PSE), which is the intensity that was perceived as more intense in 50% of the trials. Furthermore, we extracted the Just Noticeable Difference (JND), corresponding to the difference between the PSE and the intensity which is perceived stronger than the reference one in 84% of the trials.^2^

Before testing the bimodal conditions, if the unimodal conditions resulted in very different performances, either the visual or tactile ranges were scaled to be comparable (see Supplementary section 8.1 and Figure S2). Indeed, following the MLE model, the reliabilities of the two sensory cues needed to be equal to avoid the winner-take-all scenario, where one modality dominates over the other one.^2^

With these new values, the bimodal conditions were performed. Then the integrated estimate predicted from the MLE model was extracted (see Supplementary section 8.2).

Finally, we compared the accuracies between the unimodal and bimodal conditions. For both suprathreshold and subthreshold trials, we extracted for each condition the number of correct answers (i.e., strongest stimulus correctly identified).

For all the conditions, we asked subjects to report trial-by-trial whether they saw and/or felt any stimulus with above-chance confidence.

## 2.2 ERP (Event-Related Potentials) session

Participants were asked to detect the unimodal (visual or tactile) or bimodal (visuo-tactile) nature of trials (Figure 1D). They performed the task by pressing button(s) on the VR controller(s) associated with each modality (Figure 1D): subjects could press either one or both the controllers according to what stimuli they perceived. When participants were not pressing anything, it was assumed that they had not consciously perceived any stimulus: in fact, they were previously instructed to press the proper button(s) as soon as their confidence of having felt some stimulus/i was above-chance.

A total of 1050 trials was delivered in a randomized order, with an intertrial interval (ITI) jittered between 1 and 2 seconds. Suprathreshold conditions consisted of 100 V_SUPRA_, 100 T_SUPRA_, and 100 VT_SUPRA_ trials; subliminal conditions consisted of 250 V_SUB_, 250 T_SUB_, and 250 VT_SUB_ trials. Subliminal stimuli outnumbered the supraliminal ones to reduce task’s predictability and to increase the statistical power needed to analyze ERPs related to subliminal stimuli, which were expected to be smaller than those related to the supraliminal ones.^4^ In this session, supraliminal and subliminal stimuli were defined adding to, or subtracting from, respectively, the 15% to the average subjective threshold resulting from the previously described calibration procedure.^4^

*Finding the best statistical approach to analyze the EEG data recorded during the present study was challenging, as none of the few papers investigating the possible multisensory integration of couples of stimuli both administered at a subthreshold intensity involved electroencephalography. While representing a novelty in the literature, this also implied that the methods reported in previous studies about comparable topics (e.g., multisensory integration of supraliminal stimuli*^5^*, or of a subliminal versus a supraliminal stimulus*^4^*) could be only partially suitable for our purposes. It is also worth considering that the administered stimuli – even the supraliminal ones – were calibrated to have a very weak intensity (near the perceptual threshold), which adds up to the experimental conditions that make these data not perfectly comparable with (and, thus, analyzable as) those reported in the existing literature.*

*In particular, the kind of analysis used in this field (e.g., Global Field Power*^6^*, whole-scalp point-by-point analysis*^7^*) reduces data complexity in ways (e.g., “akin to spatial standard deviation”*^6^*) that are coherent with the idea of a wider and increased information communication among many distant areas as an indicator of multisensory integration*^8^*.*

*However, for the specific conditions of our study (administering stimuli calibrated to be slightly above or below the average perceptual threshold of each participant) this approach could result too conservative to catch differences between subliminal-only conditions, that – even if significant – are reasonably characterized by a smaller effect size than that of their supraliminal counterparts.*

*For these reasons, we preferred to follow a two-step approach starting with a classical ERP analysis followed by a more in-depth ICA-based ERP decomposition. The steps of each analysis are reported in the following sub-chapters and in the Method section of the manuscript.*

## *2.3 ERPs (Event-Related Potentials) analysis*

*A first analysis searched for significant differences among ERPs of unimodal and bimodal stimuli, in the time latencies ranging from the 100ms preceding each stimulus to the 400ms after it. Significant differences were considered reliable if matching the same criteria described in the Method section of the manuscript and adopted for the components shown in Figure 3: bimodal activity templates had to be significantly different from their unimodal correspondents simultaneously for at least 10ms consecutively, while the unimodal activity templates were not significantly differing from each other.*

*The application of these strict criteria resulted in the absence of significant differences worth of being interpreted as signs of multimodal integration, which does not exclude the possibility that some latencies could present significant differences in some electrodes. For the sake of exhaustivity, we report in Figure S5 the bimodal VS unimodal comparisons for some representative electrodes (i.e., OZ, CPZ, FCZ, AFZ): none of them yielded positive results, furtherly motivating us to move to a more sensitive analysis.*

*Similarly, we show more detailed and exhaustive ERPs in Figures S6, S7 and S8.*

## *2.4 ICA-based ERP decomposition*

*The ICA-based ERP decomposition modeled ERPs as the sum of temporally independent components, as detailed in the manuscript.*

*Figure S3 shows topography and activity templates of the four independent components in response to supraliminal and subliminal, unimodal and bimodal trials: as expected, these plots show many statistically significant differences between the supraliminal and the subliminal version of each kind of stimulus.*

*Figure S4 shows the same data grouped for subliminal or supraliminal stimuli, excluding the component shown in Figure 3 of the manuscript. These plots show no significant differences respecting the strict criteria detailed in the manuscript’s Methods section, which – together with a topography compatible with brain hubs of multisensory integration – corroborates the interpretation of the component shown in Figure 3 of the manuscript as the component mostly representing multisensory integration.*

## 2.5 JND analysis

For each condition, we fitted a cumulative normal probability distribution using maximum likelihood estimation, i.e., with a Generalized Linear Mixed Model (GLMM). The reference trial was used as the predictor, and a probit link function was used to obtain the psychometric functions, which represent the probability of judging the reference trial as having a higher intensity with respect to the comparison one. The Goodness of fit was assessed with the pseudo R2.^21^ For each psychometric curve we computed the Point of subjective equality (PSE), hence the intensity of the trial that was perceived as stronger than the reference in 50% of cases. Then we extracted the JND, considered the smallest difference that can be detected between two trials, as the difference between the PSE and the intensity perceived as more intense in 84% of cases.^2^ These analyses were performed with R Studio with the MyxedPsy library.^3^ From the unimodal conditions, we calculated the predicted optimal integration behavior following the MLE model (Supplementary section 8.2). These performances were compared with an Anova for multiple comparison with a Bonferroni Holm correction. The comparison of the accuracies was performed with the Kruskal Wallis test. Finally, for the accuracy in the subthreshold conditions, we generated random numbers from the binomial distribution specified by the number of trials (i.e., 800) and the probability of success for each trial fixed at 0.5. This distribution with 50% probability of success (i.e., chance level) was compared to each subthreshold condition with a Wilcoxon rank sum test.

## 2.6 Rescaling of unimodal conditions

To allow the improvement brought by the integration of the two sensory cues to be maximal, we computed the new levels of intensities of one of the two modalities from the other, according to the following equation:^22^

$$I_{i_{n}}=\left( i_{n}-{Ref}_{n} \right)\frac{{JND}_{m}}{{JND}_{n}}+I_{{Ref}_{n}}$$

Where $i_{n}$ is the intensity for the unimodal condition not to be scaled, ${Ref}_{n}$ is the reference stimulus intensity of the unimodal condition not to be scaled, ${JND}_{m}$ and ${JND}_{n}$ are the 84% discrimination thresholds for the tactile and visual cue obtained, and $I_{{Ref}_{n}}$ is the intermediary level of intensity that was selected to represent the standard stimulus.

## 2.7 Optimal integration

According to the model proposed by Ernst & Banks,^2^ the predicted performance of the bimodal condition can be derived from the visual and tactile cues as a weighted average between the individual sensory signals:

$$S_{VT}= w_{V}S_{V}+w_{T}S_{T}$$

Where $S_{VT}$ is the final integrated estimate, $S_{V}$ and $S_{T}$ are the visual and tactile cues, and $w_{V}$and $w_{T}$ are the weights of the unisensory cues and should sum up to 1 (W_V_ + W_H_ = 1) and are proportional to the reliability of the stimulus:

$$w_{V}= \frac{R_{V}}{R_{V}+ R_{H}} {and w}_{H}= \frac{R_{H}}{R_{V}+ R_{H}}$$

Where the reliability (R) is the variance, and is obtained as follows:

$$R_{i}=1/\sigma_{i}^{2}$$

Finally, the variance of the optimal MLE estimate ($\sigma_{VT}^{2}$) is obtained from the visual ($\sigma_{V}^{2}$) and tactile ($\sigma_{T}^{2}$) variance as follows:

$$\sigma_{VT}^{2}= \frac{\sigma_{V}^{2}\sigma_{T}^{2}}{\sigma_{V}^{2}+\sigma_{T}^{2}}$$

## 2.8 Goodness of fit

The goodness of fit of the psychometric curves was assessed with the pseudo R^2^ ($R_{L}^{2}$).^21^ This index is used to calculate the goodness of fit of generalized linear models. It represents the proportional reduction in the deviance, where the deviance is considered as a variation measure analogous to the one in linear regression analysis.^23^ The index is calculated as follows:

$$R_{L}^{2}= \frac{D_{null}-D_{fitted}}{D_{null}}$$

Where $D_{null}$ and $D_{fitted}$ are respectively the null deviance (difference between a model with only the intercept and a saturated model) and the model deviance (difference between a model with one predictor and the saturated model).

**Supplementary Figures**


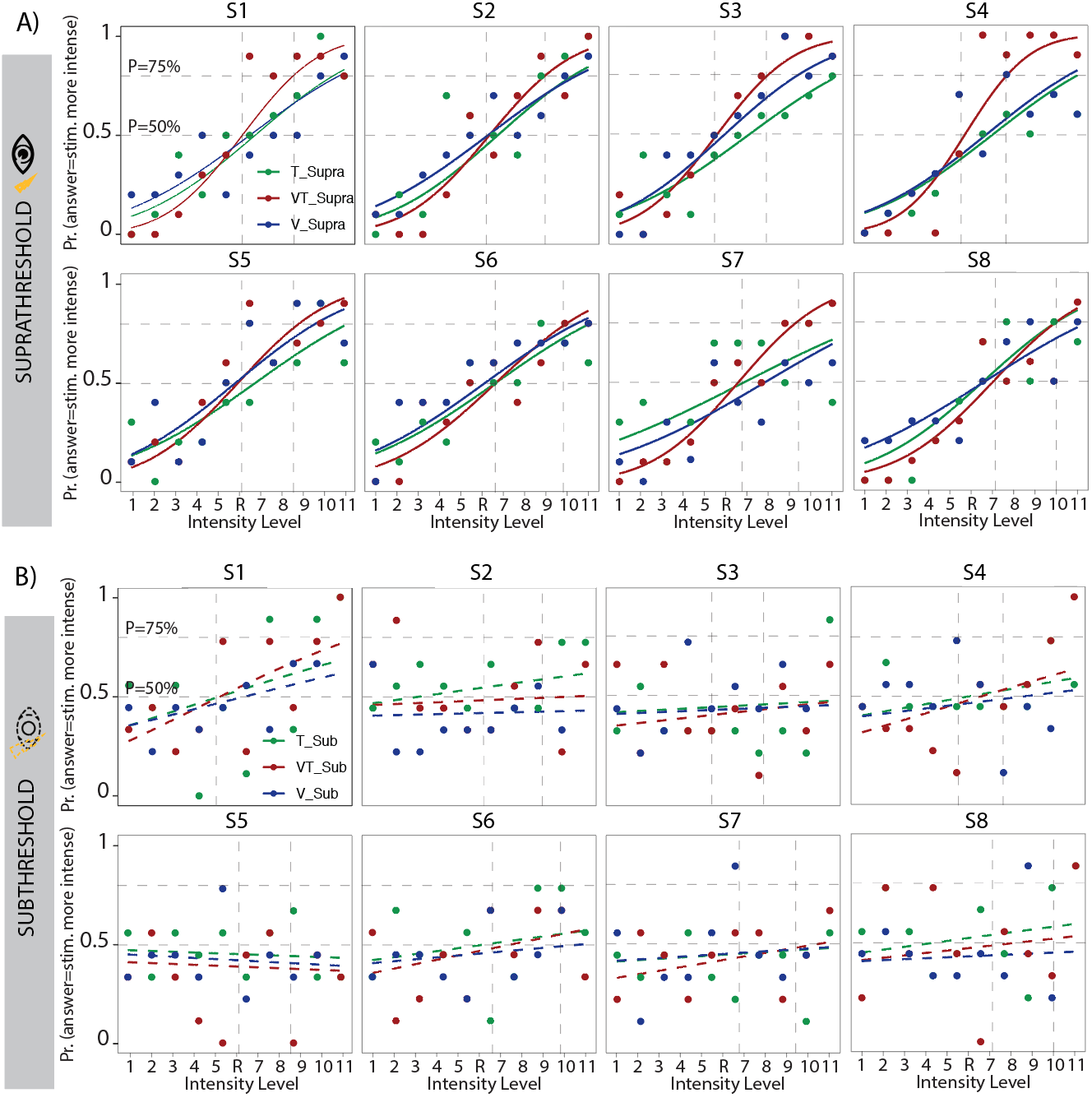


***Figure S1:*** *All Psychometric curves for the supra (A) and subthreshold (B) conditions, for all sensory modalities and all subjects: tactile (green), visual (blue), visuo-tactile (red). T=tactile; V=visual; VT=visuo-tactile; Pr=probability; stim=stimulus*


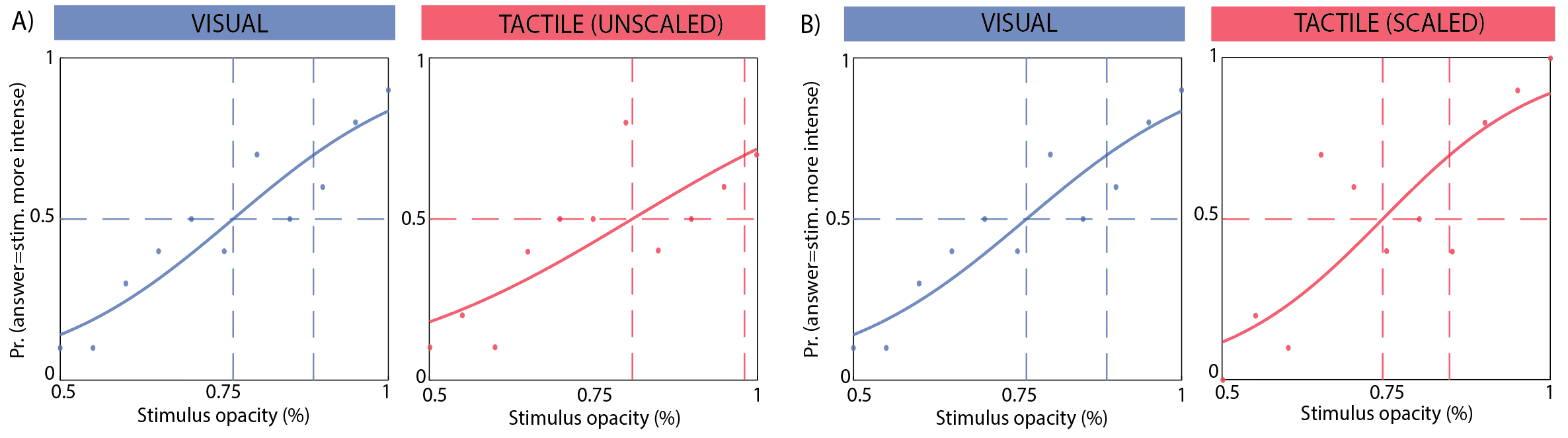


***Figure S2:*** *Rescaling of psychometric curves. A) Comparison of the original psychometric curves. The tactile curve (red, on the right) has a worse performance than the visual one (blue, on the left). B) Comparison of the new psychometric curves. The tactile curve (red, on the right) has a more similar performance compared to the visual one (blue, on the left). This allows to avoid the winner take-all model.*


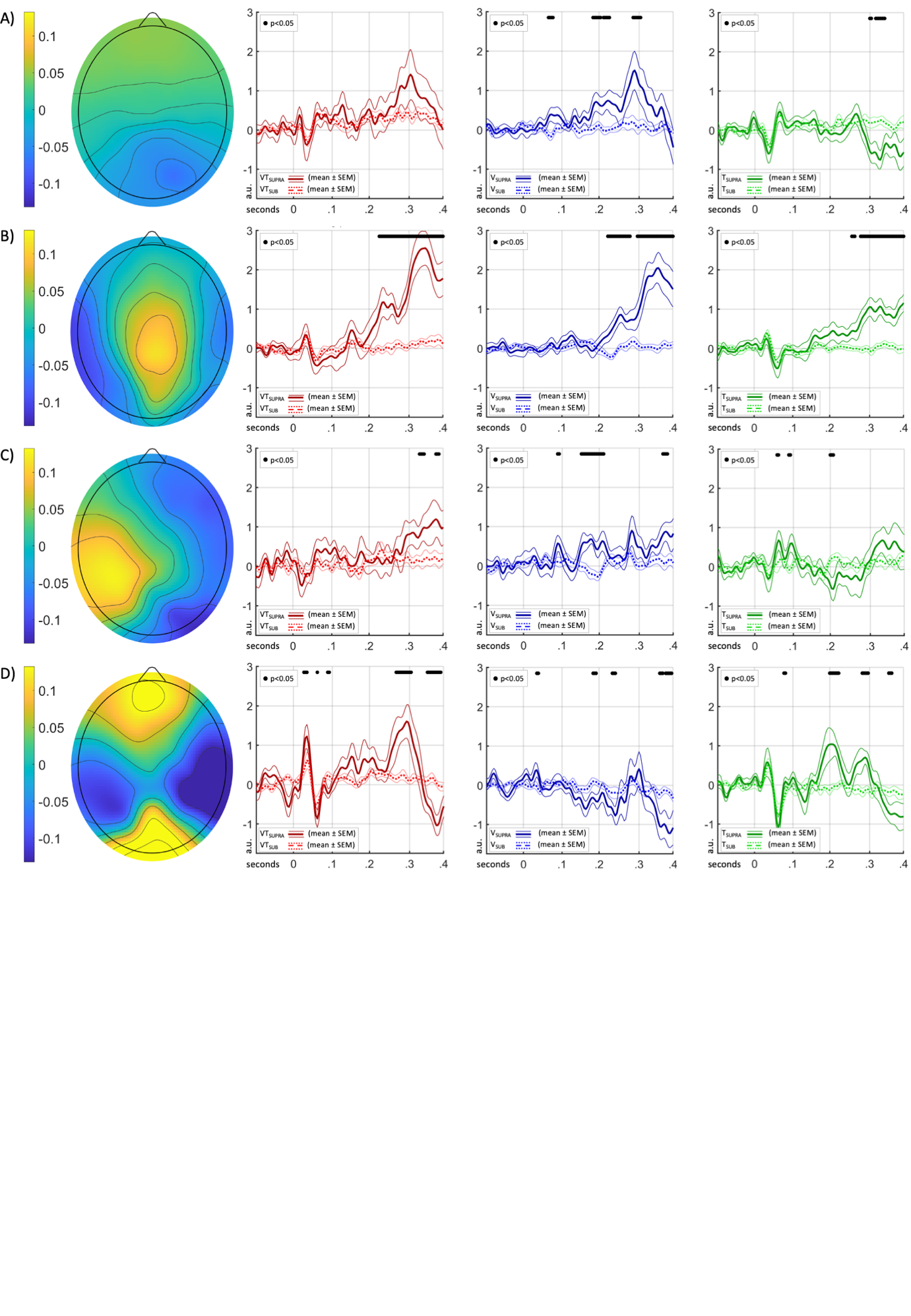


*Figure S3: Topography and activity templates of the four independent components in response to supraliminal and subliminal, unimodal and bimodal trials. The first column shows component scalp topography for each of the four components retained. The second column shows activity templates for supraliminal VS subliminal visuo-tactile trials; the second column shows the same for supraliminal VS subliminal visual trials; the third column shows activity templates for supraliminal VS subliminal tactile trials. Thicker lines (solid or dashed for supraliminal or subliminal trials, respectively) represent the group mean, thinner ones represent the group mean ± the standard error of the mean (SEM); the black dots represent latencies at which the trials produced significantly different activity templates (p<0.05); a.u. = arbitrary units.*


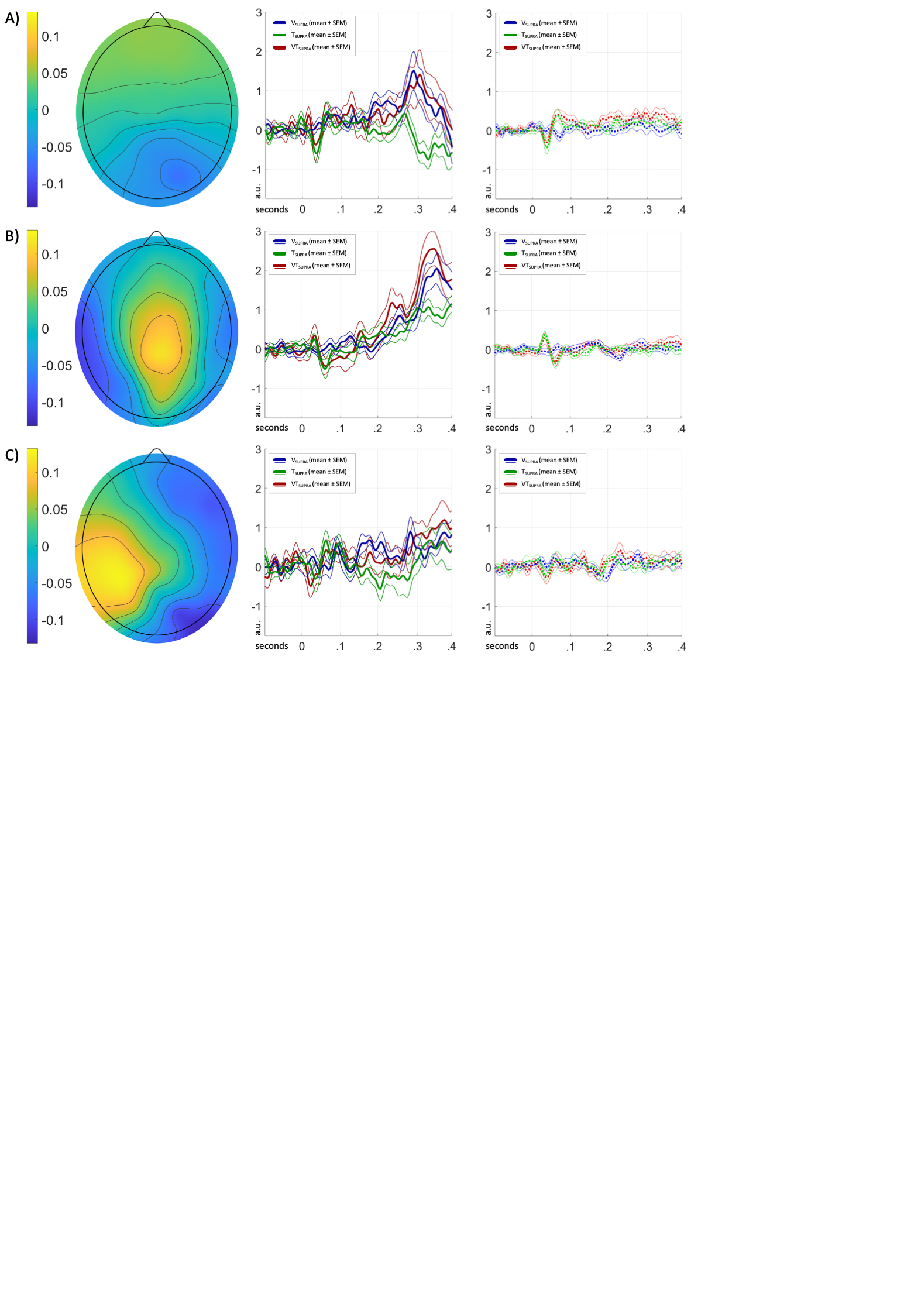


*Figure S4: Topography and activity templates of the three independent components not included in Figure 3 in response to supraliminal and subliminal, unimodal and bimodal trials. The first column component scalp topography of the three components not included in Figure 4. The second column shows activity templates for supraliminal trials; the third one shows the same for subliminal trials. Thicker lines (solid or dashed for supraliminal or subliminal trials, respectively) represent the group mean, thinner ones represent the group mean ± the standard error of the mean (SEM); note the absence of latencies at which bimodal trials significantly (p<0.05) differed from both unimodal trials simultaneously, while these latter were not significantly differing from each other; a.u. = arbitrary units.*

****** *Figure S5: ERPs related to supraliminal and subliminal, unimodal and bimodal trials for each of 4 electrodes (OZ, CPZ, FCZ, AFZ) distributed along the central axis of the scalp. Note that the vertical scale of each plot is auto-scaled to different values to fit the whole ERP. Thicker lines (solid or dashed for supraliminal or subliminal trials, respectively) represent the group mean, thinner ones represent the group mean ± the standard error of the mean.******* *Figure S6: ERPs related to supraliminal unimodal and bimodal for each electrode, with the corresponding scalp map referring to different latencies (i.e., 30ms, 60ms, 250ms, 300ms and 350ms after stimulus onset).*

* Figure S7: ERPs related to subliminal unimodal and bimodal for each electrode, with the corresponding scalp map referring to different latencies (i.e., 30ms, 60ms, 250ms, 300ms and 350ms after stimulus onset).*

****** *Figure S8: ERPs related to supraliminal and subliminal, unimodal and bimodal for each electrode represented in its position on the scalp.*

# Supplementary References

1. Cheesman J, Merikle PM. Priming with and without awareness. *Perception & Psychophysics*. 1984;36(4):387-395. doi:10.3758/BF03202793

2. Ernst MO, Banks MS. Humans integrate visual and haptic information in a statistically optimal fashion. *Nature*. 2002;415(6870):429-433. doi:10.1038/415429a

3. Balestrucci P, Ernst MO, Moscatelli A. *Psychophysics with R: The R Package MixedPsy*. Neuroscience; 2022. doi:10.1101/2022.06.20.496855

4. Nierhaus T, Forschack N, Piper S, et al. Imperceptible Somatosensory Stimulation Alters Sensorimotor Background Rhythm and Connectivity. *JNEUROSCI*. 2015;35(15):5917-5925. doi:10.1523/JNEUROSCI.3806-14.2015

5. Ronga I, Galigani M, Bruno V, et al. Seeming confines: Electrophysiological evidence of peripersonal space remapping following tool-use in humans. *Cortex*. 2021;144:133-150. doi:10.1016/j.cortex.2021.08.004

6. Noel JP, Chatelle C, Perdikis S, et al. Peri-personal space encoding in patients with disorders of consciousness and cognitive-motor dissociation. *NeuroImage: Clinical*. 2019;24:101940. doi:10.1016/j.nicl.2019.101940

7. Fossataro C, Galigani M, Rossi Sebastiano A, Bruno V, Ronga I, Garbarini F. Spatial proximity to others induces plastic changes in the neural representation of the peripersonal space. *iScience*. 2023;26(1):105879. doi:10.1016/j.isci.2022.105879

8. Tononi G. An information integration theory of consciousness. *BMC Neuroscience*. 2004;5(1):42. doi:10.1186/1471-2202-5-42

9. Junghofer M, Elbert T, Tucker DM, Rockstroh B. Statistical control of artifacts in dense array EEG/MEG studies. *Psychophysiology*. 2000;37(4):523-532. doi:10.1111/1469-8986.3740523

10. Bell AJ, Sejnowski TJ. An Information-Maximization Approach to Blind Separation and Blind Deconvolution. *Neural Computation*. 1995;7(6):1129-1159. doi:10.1162/neco.1995.7.6.1129

11. Makeig S, Bell A, Jung TP, et al. Advances in neural information processing systems. *Independent component analysis of electroencephalographic data*. 1996;8:145-151.

12. Pion-Tonachini L, Kreutz-Delgado K, Makeig S. ICLabel: An automated electroencephalographic independent component classifier, dataset, and website. *NeuroImage*. 2019;198:181-197. doi:10.1016/j.neuroimage.2019.05.026

13. Piarulli A, Menicucci D, Gemignani A, et al. Likeness-Based Detection of Sleep Slow Oscillations in Normal and Altered Sleep Conditions: Application on Low-Density EEG Recordings. *IEEE Trans Biomed Eng*. 2010;57(2):363-372. doi:10.1109/TBME.2009.2031983

14. Driver J, Noesselt T. Multisensory Interplay Reveals Crossmodal Influences on ‘Sensory-Specific’ Brain Regions, Neural Responses, and Judgments. *Neuron*. 2008;57(1):11-23. doi:10.1016/j.neuron.2007.12.013

15. Hidaka S, Teramoto W, Sugita Y. Spatiotemporal Processing in Crossmodal Interactions for Perception of the External World: A Review. *Front Integr Neurosci*. 2015;9. doi:10.3389/fnint.2015.00062

16. Laurino M, Menicucci D, Piarulli A, et al. Disentangling different functional roles of evoked K-complex components: Mapping the sleeping brain while quenching sensory processing. *NeuroImage*. 2014;86:433-445. doi:10.1016/j.neuroimage.2013.10.030

17. Jung TP, Makeig S, McKeown MJ, Bell AJ, Lee TW, Sejnowski TJ. Imaging brain dynamics using independent component analysis. *Proc IEEE*. 2001;89(7):1107-1122. doi:10.1109/5.939827

18. Himberg J, Hyvärinen A, Esposito F. Validating the independent components of neuroimaging time series via clustering and visualization. *NeuroImage*. 2004;22(3):1214-1222. doi:10.1016/j.neuroimage.2004.03.027

19. Menicucci D, Artoni F, Bedini R, et al. Brain Responses to Emotional Stimuli During Breath Holding and Hypoxia: An Approach Based on the Independent Component Analysis. *Brain Topogr*. 2014;27(6):771-785. doi:10.1007/s10548-013-0349-z

20. Bocharov AV, Savostyanov AN, Tamozhnikov SS, et al. Oscillatory dynamics of perception of emotional sentences in healthy subjects with different severity of depressive symptoms. *Neuroscience Letters*. 2020;728:134888. doi:10.1016/j.neulet.2020.134888

21. Cohen P, Cohen P, West SG, Aiken LS. *Applied Multiple Regression/Correlation Analysis for the Behavioral Sciences*. 0 ed. Psychology Press; 2014. doi:10.4324/9781410606266

22. Risso G, Valle G, Iberite F, et al. Optimal integration of intraneural somatosensory feedback with visual information: a single-case study. *Scientific Reports*. 2019;9(1). doi:10.1038/s41598-019-43815-1

23. An Introduction to Logistic Regression Diagnostics. In: *Applied Logistic Regression Analysis*. SAGE Publications, Inc.; 2002:68-91. doi:10.4135/9781412983433.n4
